# Lack of Type 2 Innate Lymphoid Cells Promotes a Type I-Driven Enhanced Immune Response in Contact Hypersensitivity

**DOI:** 10.1016/j.jid.2018.03.001

**Published:** 2018-09

**Authors:** David A. Rafei-Shamsabadi, Saskia van de Poel, Britta Dorn, Stefanie Kunz, Stefan F. Martin, Christoph S.N. Klose, Sebastian J. Arnold, Yakup Tanriver, Karolina Ebert, Andreas Diefenbach, Timotheus Y.F. Halim, Andrew N.J. McKenzie, Thilo Jakob

**Affiliations:** 1 Allergy Research Group, Department of Dermatology, Medical Center-University of Freiburg, Faculty of Medicine, University of Freiburg, Freiburg, Germany; 2 Experimental Dermatology and Allergy Research Group, Department of Dermatology and Allergology, University Medical Center Giessen and Marburg, Campus Giessen, Justus Liebig University, Giessen, Germany; 3 Institute of Medical Microbiology and Hygiene, University Medical Center, Freiburg, Germany; 4 Institute of Experimental and Clinical Pharmacology and Toxicology, Faculty of Medicine, University of Freiburg, Freiburg, Germany; 5 Department of Microbiology, Charité-University Medical Centre Berlin, Berlin, Germany; 6 Medical Research Council, Laboratory of Molecular Biology, Cambridge, Cambridgeshire, UK

**Keywords:** CHS, contact hypersensitivity, ILC, innate lymphoid cell, LN, lymph node, MHC, major histocompatibility complex, NK, natural killer, Th, T helper, TNCB, 2,4,6-Trinitrochlorobenzene, TNF, tumor necrosis factor

## Abstract

Allergic contact dermatitis and its animal model, contact hypersensitivity, are T-cell-mediated inflammatory skin diseases that require activation of the innate immune system. Here we investigate the role of innate lymphoid cells (ILCs) during the elicitation phase of 2,4,6-trinitrochlorobenzene-induced contact hypersensitivity using *Eomes*^Gfp/+^ x *Rorc(γt)-*Cre^Tg^ x *Rosa26R*^Yfp/+^ reporter mice. Ear swelling responses, cutaneous ILC numbers, and cytokine production were determined at different time points. Functional analyses were performed in a CD90.1/.2 congenic adoptive transfer model that allowed selective antibody-mediated depletion of ILCs before hapten challenge, and in *Rora*^sg/flox^*Il7r*^Cre/+^ mice, which lack ILC2. Hapten challenge induced early increases of natural killer cells in skin and ear draining lymph nodes corresponding to the peak ear swelling response. In contrast, ILC1, 2, and 3 showed a delayed increase in numbers corresponding to the contact hypersensitivity resolution phase. Hapten challenge induced increased marker cytokines in all ILC subtypes and an activated phenotype in ILC2. Depletion of all ILC resulted in a significantly enhanced ear swelling response. Similarly, ILC2-deficient mice (*Rora*^sg/flox^*Il7r*^Cre/+^) displayed increased ear swelling responses on hapten challenge, suggesting that ILC2 act as negative regulators in the type 1-dominated immune response of contact hypersensitivity.

## Introduction

Allergic contact dermatitis is a prevalent inflammatory skin disease triggered by low-molecular-weight organic chemicals or metal ions that penetrate the skin and bind covalently or by complex formation to proteins, thereby activating the innate and adaptive immune response. In the mouse model of allergic contact dermatitis, the contact hypersensitivity (CHS) model, hapten-specific CD8^+^ cytotoxic T cells have been demonstrated to be the key effector cells in the elicitation phase rendering CHS a classical type 1-driven adaptive immune response (Fyhrquist et al., 2012). In addition, we and others have previously demonstrated that sensing of danger signals by cells of the innate immune system including dendritic cells, neutrophils, and mast cells represents a crucial element in the initiation and elicitation of CHS responses (Dudeck et al., 2011, Esser et al., 2012, Martin et al., 2008, Weber et al., 2010, Weber et al., 2015). Over recent years, a family of heterogeneous innate immune cells of the lymphoid lineage have been identified and classified as innate lymphoid cells (ILCs). In analogy to T-cell subsets, ILCs can be classified into three helper-like ILC groups (ILC1, ILC2, and ILC3) and one killer ILC group (natural killer [NK] cells) based on the developmental dependence on transcription factors and expression of marker cytokines (Artis and Spits, 2015, Eberl et al., 2015). Previous studies showed that ILCs are present in the skin and have suggested that ILCs mediate pathology in a mouse model of atopic dermatitis (Kim et al., 2013) as well as in psoriatic plaque formation (Pantelyushin et al., 2012). Group 2 ILC are induced in the wounded skin of mice and humans and promote wound healing in an IL33-dependent manner (Rak et al., 2016). In addition, they promote type 2-driven immune responses by supporting T helper type 2 (Th2) differentiation of naïve CD4^+^ T cells through production of type 2 cytokines, for example, IL5 and IL13, and by expression of major histocompatibility complex (MHC) class II on their cell surface allowing further T-cell priming (Christianson et al., 2015, Oliphant et al., 2014).

The role of different ILC subsets in CHS responses has not been addressed so far. In this study, we quantified all groups of ILCs in the ear skin and ear draining lymph nodes (LN) using *Eomes*^Gfp/+^ x *Rorc(γt)-*Cre^Tg^ x *Rosa26R*^Yfp/+^ double reporter mice (from now on referred to as EOMES^Gfp^RORγt-fate map [fm] mice) under steady-state conditions and in a 2,4,6-trinitrochlorobenzene (TNCB)-elicited CHS model. In sensitized mice, NK cell numbers increased early after allergen challenge in ear skin. In contrast, ILC1, ILC2, and ILC3 showed a delayed increase in the skin that coincided with the resolution phase of the CHS response. Dermal ILC2 represented the most prominent population and displayed an activated phenotype. Antibody-mediated depletion of all ILCs leads to a dramatic increase in ear swelling responses, and similarly, mice with an ILC2 deficiency displayed enhanced ear swelling responses. Taken together, these data support the concept of ILC2 as possible counter-regulators of the type 1-driven immune response of hapten-mediated CHS.

## Results

### ILC2 are the predominant ILC subset in the ear skin of naïve mice

Using EOMES^Gfp^ RORγt-fm double reporter mice, we were able to identify NK cells and all three ILC groups in ear skin and ear draining LNs of naïve mice (for gating strategy and definition of ILC subtypes, see Supplementary Figure S1a online and Supplementary Methods online). Under steady-state conditions, ILC2 represent the predominant ILC subset in the skin followed by ILC3 and by far fewer ILC1 and NK cells (Supplementary Figure S2a online). In contrast, NK cells are the most frequent ILC subtype in the ear draining LNs of naïve mice with much lower numbers of ILC1, ILC2, and ILC3 (Supplementary Figure S2b). Lineage negative, CD127^+^, RORγt^–^, EOMES^–^, NCR^–^, ICOS^+^, and CD25^+^ ILC2 in the ear skin predominantly expressed CD103 (Supplementary Figure S1b), which confirmed their classification as “dermal ILC2” as previously described by Roediger et al. (2013). We conclude that ILC2 and ILC3 represent the dominant resident innate lymphoid cell populations under steady-state conditions in the skin.

### On hapten challenge NK cells increase in ear skin before ILC2 and ILC3

**Figure 1:**
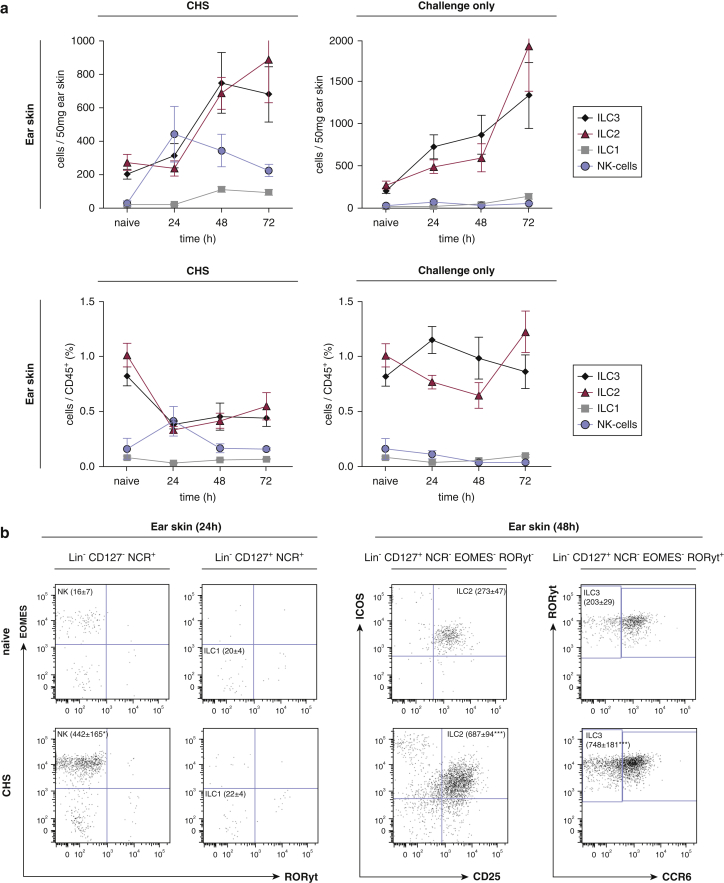

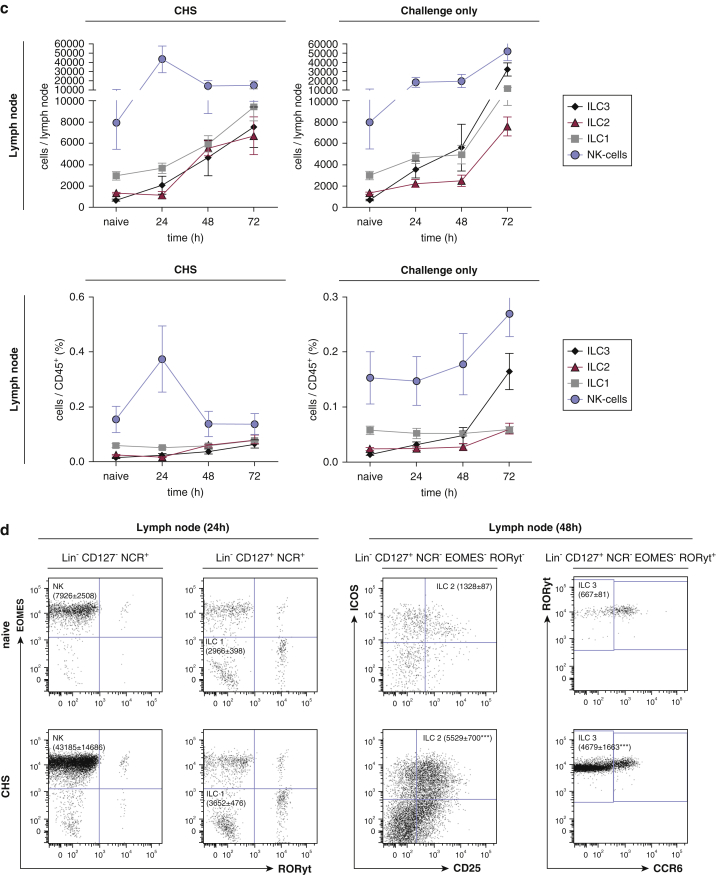
**On hapten challenge NK cells increase in ear skin before ILC2 and ILC3.** (**a**, **c**) Kinetics of absolute cell numbers (upper panel) and relative cell numbers (lower panel) for all ILC subsets in the (**a**) ears and (**c**) lymph nodes of mice sensitized and challenged with TNCB (CHS) (left graphs) and mice only challenged with TNCB (challenge only) (right graphs). (**b**, **d**) Representative concatenate dot plots visualizing changes in numbers of NK cells and ILC1 at 24 hours and ILC2 and ILC3 at 48 hours, respectively, in CHS mice compared with naïve mice. Values are shown as absolute cell numbers per 50 mg ear skin and percentages of living CD45^+^ leukocytes, respectively. Data are shown as mean ± standard error of the mean, pooled data from three independent experiments with at least n ≥ 5 mice per group. Concatenate dot plots show data of pooled samples of naive and CHS groups of mice, respectively (n ≥ 5 mice per group). *P < 0.05, **P < 0.01, ***P < 0.001, and ****P < 0.0001. EOMES^Gfp^ RORγt-fm mice were used for these experiments. CHS, contact hypersensitivity; EOMES, eomesodermin; NCR, natural cytotoxicity triggering receptor, that is, NK1.1 and NKp46; NK, natural killer; ILC, innate lymphoid cell; TNCB, 2,4,6-trinitrochlorobenzene.

To evaluate changes in ILC numbers during the elicitation phase of CHS, ILC numbers were analyzed in ear skin and ear draining LNs (Figure 1) of EOMES^Gfp^ RORγt-fm double reporter mice at different time points after TNCB challenge. Hapten challenge in sensitized mice induced an increase in NK cell numbers that peaked at 24 hours and subsequently decreased paralleling the kinetics of the ear swelling response (Figure 1a, left graph, upper panel). In contrast, ILC2 and 3 showed a delayed and prolonged increase beginning between 24 and 48 hours after challenge. There were fewer ILC1 in ear skin, but they also increased between 24 and 48 hours after hapten challenge. Hapten challenge in nonsensitized mice (challenge only) induced a different pattern, which was dominated by a continuous increase in ILC2 and ILC3, whereas NK cells and ILC1 did not increase in numbers and were barely detectable (Figure 1a, right graph, upper panel). Figure 1b and d show representative dot plots of different groups of ILC in ear skin and ear draining LNs of naive and sensitized mice at 24 and 48 hours after hapten challenge. Percentages of ILCs within the CD45^+^ leucocyte population were analyzed (Figure 1a and c, lower panel). In the CHS group, percentages of ILC2 and 3 in the skin show an early decrease at 24 hours and a subsequent slight increase at 48 and 72 hours. In contrast, percentages of NK cells significantly rise 24 hours after allergen challenge and decrease at 48 and 72 hours.

Similar changes in cell populations were observed in ear draining LNs (Figure 1c and d), the only major difference being an early and prolonged increase of NK cells in nonsensitized mice that were hapten challenged (challenge only). Hapten challenge induced an increase in total and relative ILC2 and 3 cell numbers starting at 48 hours with even higher numbers at 72 hours regardless of whether mice were sensitized or not (Figure 1c and d). Taken together, NK cells seemed to represent the major cell type of the early inflammatory response in CHS paralleling the highest ear swelling response, whereas ILC2 and ILC3 numbers most prominently increased during the resolution phase of CHS.

### Innate lymphoid cells produce their respective marker cytokines in skin and ear draining LNs during the elicitation phase of CHS

**Figure 2:**
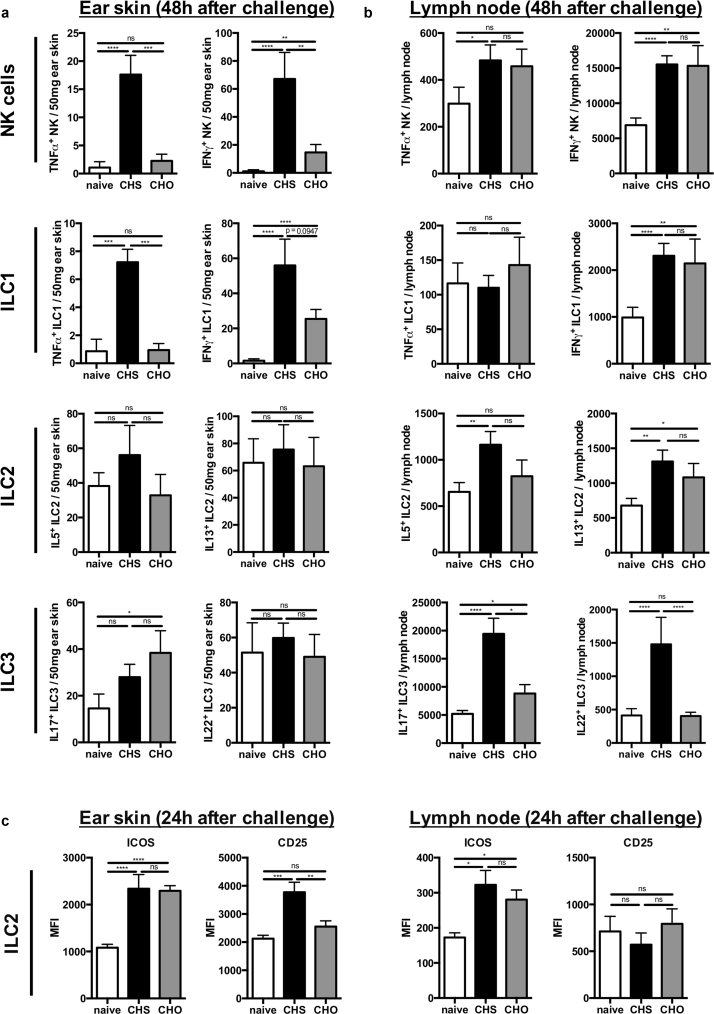
**Cytokine expression by ILC in ear skin and ear draining lymph nodes during the elicitation phase of CHS.** Cytokine production of all ILC subsets in the (**a**) ear skin and (**b**) ear draining lymph nodes at 48 hours after antigen challenge in CHS and challenge-only mice compared with naïve mice. (**c**) ICOS and CD25 expression of ILC2 isolated from ear skin (**c**, left graphs) and ear draining lymph nodes (**c**, right graphs) at 24 hours after allergen challenge in CHS and challenge-only mice. Values are shown as absolute cell numbers per 50 mg ear skin and per total ear draining lymph node, respectively. Data are shown as mean ± standard error of the mean, pooled data of three independent experiments with n ≥ 5 mice per group. *P < 0.05, **P < 0.01, ***P < 0.001, and ****P < 0.0001. EOMES^Gfp^ RORγt-fm mice were used for these experiments. CHO, challenge only; CHS, contact hypersensitivity; EOMES, eomesodermin; ICOS, inducible T-cell costimulator; ILC, innate lymphoid cell; MFI, mean fluorescence intensity; NCR, natural cytotoxicity triggering receptor; NK, natural killer; ns, not significant; TNF, tumor necrosis factor.

Next, we assessed the cytokine production of the different ILC subsets during the elicitation phase of CHS. Hapten challenge in sensitized mice induced markedly increased numbers of IFNγ and tumor necrosis factor (TNF)-positive NK cells in the skin compared with naïve and challenge-only mice indicating a proinflammatory response profile (Figure 2a). A similar pattern was observed for skin ILC1 with increased IFNγ and TNF production (Figure 2a). However, changes in IL13 and IL5 in ILC2, and IL17 and IL22 in ILC3, did not reach statistical significance, and similar increases were seen in mice that were hapten challenged only (Figure 2a). Analogous changes were observed in ear draining LNs: TNF and IFNγ production of NK cells was markedly increased in hapten challenged compared with naïve mice (Figure 2b). Similarly, hapten challenge induced significantly higher IL5 and IL13 production in ILC2 and IL17 and IL22 production in ILC3 as compared with naïve mice (Figure 2b). Furthermore, CD103^+^ ILC2 in the skin showed a significant increase in inducible T-cell costimulator and CD25 expression in sensitized mice 24 hours after hapten challenge (Figure 2c, left panel), suggesting an activated phenotype of dermal ILC2 (Paclik et al., 2015). Finally, inducible T-cell costimulator but not CD25 expression was significantly increased in ILC2s of the ear draining LNs (Figure 2c, right panel).

### Depletion of all ILC subsets leads to an enhanced ear swelling response

**Figure 3:**
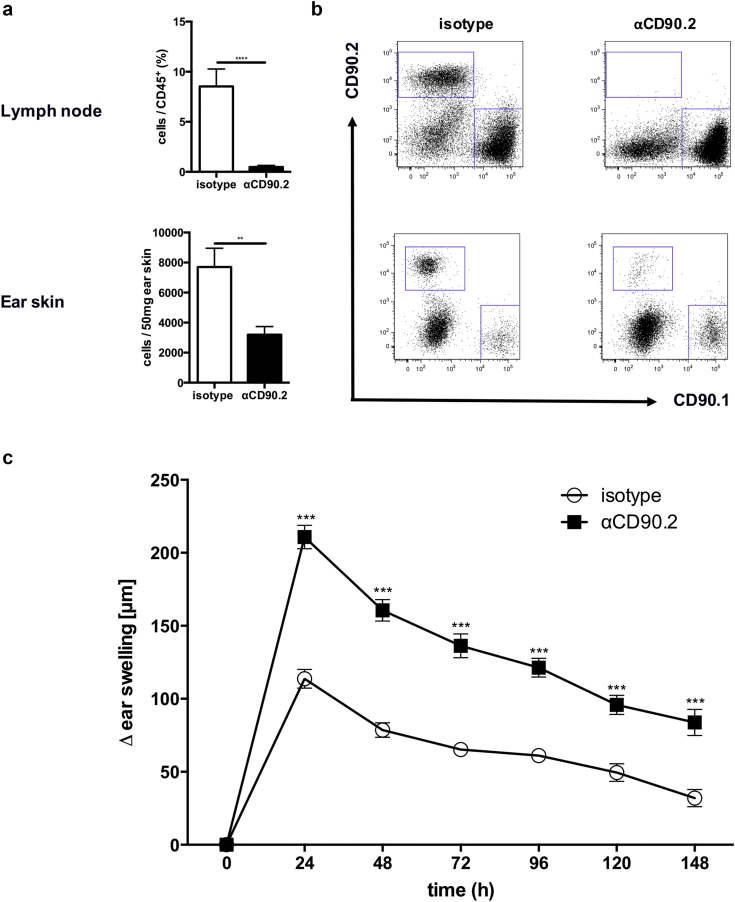
**Depletion of all ILC subsets leads to an enhanced ear swelling response.** (**a**) Verification of ILC depletion in ear draining lymph nodes (**a**, upper panel) and ear skin (**a**, lower panel) after administering anti-CD90.2 (200 μg) every other day (4 days total) to CD90.2 *Rag1*^–/–^ recipient mice that were reconstituted with FACS-sorted CD3^–^, CD8^+^, and CD4^+^ T cells from CD90.1^+^ donor mice. Values in (**a**) are displayed as absolute cell numbers per 50 mg ear skin and percent age of cells of total leukocytes in the ear draining lymph nodes, respectively. (**b**) Representative dot plots showing depletion of CD90.2^+^ leukocytes in ear draining lymph nodes (**b**, upper panel) and ear skin (**b**, lower panel). (**c**) Ear swelling response after the last allergen challenge in ILC-depleted versus isotype-treated mice shown as delta from baseline ear thickness. Data in (**a**) and (**c**) are shown as mean ± standard error of the mean, pooled data of three independent experiments with n ≥ 5 mice per group. *P < 0.05, **P < 0.01, ***P < 0.001, and ****P < 0.0001. ILC, innate lymphoid cell.

To determine whether ILCs play a functional role during the elicitation phase of contact hypersensitivity, we used an adoptive transfer model for the TNCB-based contact allergy in congenic mice (adoptive transfer of CD90.1 T cells into CD90.2 *Rag1*^*–/–*^ mice) (as described in the Methods section) that allowed the selective depletion of autochthonous ILCs by targeting CD90.2. Effective ILC depletion was confirmed by flow cytometry in skin draining LNs and to a lesser extent in ear skin (Figure 3a and b). Mice that had undergone ILC depletion displayed a significantly increased ear swelling response compared with isotype-treated controls that lasted over 6 days and did not return to baseline levels (Figure 3c). Analysis of T-cell infiltrates in the ear tissue demonstrated enhanced numbers of T-bet and Foxp3 expressing CD4^+^ T cells under ILC-depleted conditions as compared with isotype control, whereas no significant differences in numbers of GATA3 and RORγt expressing CD4^+^ T cells were observed (Supplementary Figure S3 online). In draining LNs, no significant changes in the analyzed T-cell subsets were detected (Supplementary Figure S3). Thus, selective depletion of ILC in sensitized mice before hapten challenge resulted in an enhanced inflammatory response with a shift toward a type 1 phenotype, suggesting a regulatory role of ILCs in the elicitation phase of CHS.

### Lack of ILC2 leads to increased CHS responses

**Figure 4:**
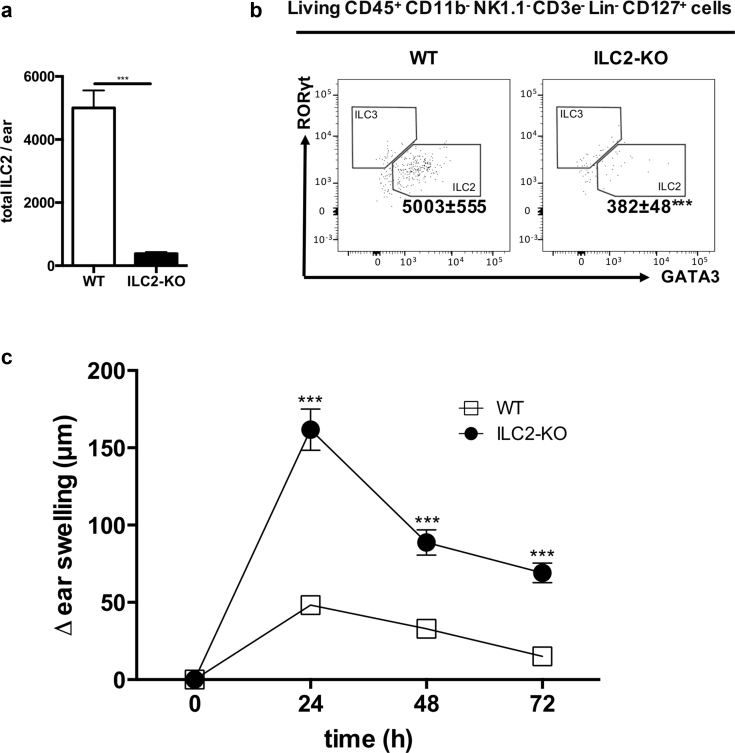
**Lack of ILC2 leads to increased CHS responses.** (**a**) Number of ILC2 in the skin of ILC2-KO mice compared with wild-type mice. (**b**) Representative dot blots of ILC2 in the ear skin of naïve and ILC2-KO mice. (**c**) Ear swelling response of ILC2-KO compared with wild-type mice up to 72 hours after allergen challenge displayed as delta from baseline ear thickness. Values in (**a**) are displayed as absolute cell numbers per 50 mg ear skin. Data in (**b**) and (**c**) are shown as mean ± standard deviation, pooled data of two independent experiments with at least n ≥ 5 mice per group. *P < 0.05, **P < 0.01, ***P < 0.001, and ****P < 0.0001. ILC2 were first gated on live, CD45^+^ CD11b^–^ NK1.1^–^ CD3e^–^, and then lineage (CD19, B220, CD5, Gr-1, FreR1a, Ter119, F4/80)^–^ and CD127^+^. CHS, contact hypersensitivity; ILC, innate lymphoid cell; KO, knockout; NK, natural killer; WT, wild type.

To investigate the functional relevance of ILC2s in CHS, we used *Rora*^sg/flox^*Il7r*^Cre/+^ mice that lack the transcription factor RORα in all cells that express the IL7 receptor. These mice display normal Th2 function but have greatly impaired ILC2 immune responses (Oliphant et al., 2014). In line with this, these mice displayed >90% diminished ILC2 numbers in the ear skin (Figure 4a and b). *Rora*^sg/flox^*Il7r*^Cre/+^ mice were sensitized and challenged with TNCB as described above, and ear thickness was measured up to 3 days after allergen challenge. The lack of ILC2 leads to an increased ear swelling response compared with wt control mice at all time points after challenge (Figure 4c), further supporting a regulatory role of ILC2 in CHS.

## Discussion

The involvement of innate lymphoid cells, especially NK cells, in the pathogenesis of allergic contact dermatitis was proposed more than 10 years ago (O’Leary et al., 2006, Peng et al., 2013, Tang et al., 2016). In the meantime, the field of innate lymphoid cells has rapidly expanded (Artis and Spits, 2015, Eberl et al., 2015) and new tools have become available to analyze the involvement of different ILC populations in various disease models. Selected ILC populations have been reported to contribute to the pathogenesis of inflammatory skin diseases, such as ILC2 in atopic dermatitis and ILC3 in psoriasis (Batista et al., 2013, Imai et al., 2013, Kim et al., 2013, Pantelyushin et al., 2012, Salimi et al., 2013, Teunissen et al., 2014, Villanova et al., 2014). However, these skin diseases markedly differ in the immune response pattern compared with allergic contact dermatitis, and until now no data on the involvement of ILC other than NK cells are available. We therefore set out to analyze numerical kinetics of ILC subsets during the elicitation phase of CHS using EOMES^Gfp^ RORγt-fm double reporter mice that allow the robust analysis of all ILC subtypes.

NK cell numbers were significantly increased in the skin 24 hours after challenge as well as their expression of type I cytokines, namely IFNγ and TNF. These results are in line with previous reports on the role of NK cells during the elicitation phase of CHS (Carbone et al., 2010, O’Leary et al., 2006). In addition, we identified elevated numbers of ILC1 in the skin starting at 48 hours after allergen challenge that displayed prominent IFNγ and TNF production suggesting a proinflammatory contribution to this type 1-driven immune response. Similarly, intraepithelial ILC1 have been demonstrated in patients with Crohn’s disease and have been suggested to contribute as a proinflammatory IFNγ-producing population to the pathology in an anti-CD40-induced colitis model in mice (Fuchs et al., 2013).

Under steady-state conditions, dermal ILC2 represented the most prominent ILC population in the skin. On hapten challenge, we observed a delayed increase of dermal ILC2 that coincided with the resolution phase of the CHS response. Dermal ILC2 displayed an activated phenotype as demonstrated by enhanced inducible T-cell costimulator expression and production of type II signature cytokines, namely IL13 and IL5, suggesting an active contribution during the CHS response. ILC2 have been described to be necessary for worm expulsion in a *Nippostrongylus brasiliensis* infection mouse model (Hoyler et al., 2012, Moro et al., 2010, Neill et al., 2010, Oliphant et al., 2014), and to play a proinflammatory role in allergic inflammatory diseases such as allergic asthma or atopic dermatitis (Halim et al., 2016, Kim et al., 2013). To our current knowledge, no data exist that address the role of ILC2 in a type 1-driven immune response, such as the TNCB-induced CHS model. The observation that in contrast to NK cells, ILC2 numbers in the skin (and in the ear draining LNs) increased during later time points of the CHS response prompted us to speculate that ILC and in particular ILC2 may contribute to the resolution phase of the CHS response.

Migration and tissue localization of ILCs is being discussed widely and is somewhat contentious. Parabiosis experiments have demonstrated that most ILCs do not migrate from epithelial tissues to the blood or LNs in adult mice but rather proliferate in situ. In addition, there is increasing evidence that ILCs may be regenerated from local tissue-specific progenitors that populate the organ site early during embryogenesis (Bando et al., 2015, Gasteiger et al., 2015). However, it has also been shown that ILCs can acquire homing receptors for secondary lymphoid and nonlymphoid organs and thus may potentially be capable of active migration (Kim et al., 2016). On the basis of the above, we assume that the ILC subtypes identified in skin and skin draining LNs represent different cell populations. However, both populations may influence each other indirectly via cytokine production and priming of T cells, which then circulate between skin and LNs (Halim et al., 2016).

To address the functional role of ILCs during the CHS response, two different approaches were used: (i) antibody-mediated depletion of autochthonous ILC in an adoptive transfer model of hapten-sensitized T cells into *Rag1*^*–/–*^ mice and (ii) analysis of CHS responses in mice that selectively lack ILC2 (*Rora*^sg/flox^*Il7r*^Cre/+^mice) (Oliphant et al., 2014).

In the first approach, we transferred CD3^+^ CD4^+^ and CD8^+^ T cells of hapten-sensitized mice (CD90.1) into congenic *Rag1*^*–/–*^ mice (CD90.2). Before hapten challenge, autochthonous ILCs (CD90.2) were selectively depleted, which lead to significantly enhanced and long-lasting ear swelling responses. There has been some debate as to the efficacy of antibody-mediated depletion of either T cells in human skin (Clark et al., 2012) or dermal ILC2 in mouse skin (Roediger et al., 2013). Clark et al. suggested that ineffective T cell depletion could be due to a cell type critical for T cell depletion that is not present in the skin. They provide good evidence in mice that T cell depletion is dependent on the presence of neutrophils because depletion of neutrophils abrogated effective depletion. Because the presence of neutrophils is a hallmark of skin inflammation in CHS (Weber et al., 2015), we speculate that anti-CD90.2-mediated ILC depletion could also be facilitated by the presence of neutrophils in the skin. Because recipient animals in our adoptive transfer experiments have received a skin sensitization before depletion, this might contribute to effective depletion. In addition, differences in dose and frequency of application may have contributed to a more effective depletion of ILCs in the skin in our study as compared with previous reports (Roediger et al., 2013).

The ear infiltrate of ILC depleted mice showed a tendency toward a more type I biased immune response indicated by increased numbers of T-bet^+^ CD4^+^ T cells. These data support the concept of some kind of counter-regulatory role for ILC in CHS. In addition, increased numbers of Foxp3^+^ CD4^+^ T cells were observed. At the moment, it can only be speculated that this might result from a counter-regulatory mechanism to compensate for the lack of regulatory ILC2s. Functional studies will have to address the mutual cross-regulation of Foxp3^+^ T cells and ILC in the skin.

ILC2 and ILC3 express MHCII molecules on their surface and can act as antigen-presenting cells for Th cells (Hepworth et al., 2013, Hepworth et al., 2015, Oliphant et al., 2014). At least in the case of ILC3, MHCII expression has been reported to be crucial for dampening T effector cell functions against commensal bacteria in the gut, because the lack of MHCII on ILC3 resulted in an enhanced inflammatory response of commensal bacteria-specific CD4^+^ T cells. This observation supports the counter-regulatory potential of ILCs (Hepworth et al., 2013, Hepworth et al., 2015). The detailed analysis of MHCII expression on ILC2s (Supplementary Figure S4 online) revealed that in skin draining LN approximately 50% of the ILC2s express MHCII, whereas in the skin only approximately 3% express MHCII. Depletion of ILC by CD90.2 leads to a significant reduction of MHCII-positive ILC2s both in skin and LN. Currently, we can only speculate that ILC2 might regulate effector T cells in a direct fashion via MHCII. In line with this, Oliphant et al. recently demonstrated that MHCII expression on ILC2 and subsequent antigen presentation to CD4^+^ T cells is crucial for successful helminth expulsion in mice (Oliphant et al., 2014). The crosstalk between ILC2s and CD4^+^ T cells seems to require IL-2. Thus, the lack of ILC2 may lead to a higher availability of IL-2 for proliferation of other effector cells and thus lead to an augmented response in CHS.

On the basis of previous reports on the role of NK cells in CHS, we performed pilot experiments in IL-15-deficient mice, which lack NK cells (Kennedy et al., 2000). In line with previous data, IL-15-deficient mice displayed reduced hapten induced ear swelling responses, supporting the concept that NK cells contribute as effector cells in CHS (Supplementary Figure S5 online). Of note, IL15*-*deficient mice are not only selective NK cell mutants but also display marked reductions in numbers of thymic and peripheral NK T cells, memory phenotype CD8^+^ T cells, and distinct subpopulations of intestinal intraepithelial lymphocytes (Kennedy et al., 2000), which may also contribute to the observed changes. The analysis of the different ILC populations in the ear skin of IL-15-deficient mice revealed the lack of NK cells (as expected) and increased numbers of ILC2 and ILC3 under steady-state and under CHS conditions (Figure S5b and c). This increase in ILC2 and ILC3 in the absence of NK cells may reflect a compensatory mechanism suggesting a mutual cross-regulation of different ILC subsets in the skin.

Because ILC2 represented the predominant ILC subpopulation in the skin and showed an activated phenotype and increased IL13 and IL5 expression after TNCB challenge, we sought to investigate the TNCB model under ILC2-deficient conditions. In *Rora*^sg/flox^*Il7r*^Cre/+^ mice that lack ILC2 but express normal T cell and NK cell numbers that are functionally unaffected (Oliphant et al., 2014), we similarly observed an enhanced ear swelling response, which suggested that ILC2 may act as counter-regulators in the type I-dominated CHS response.

Earlier reports showing that IL-13^–/–^ mice display enhanced ear swelling responses to the hapten DNFB (Herrick et al., 2003) further support our observation. At the time, this finding was interpreted as a lack of Th2-mediated suppression (Herrick et al., 2003). Today ILC2 are known to be the major source of IL-13 production, thus playing a crucial role in innate immune responses to worms and inhaled allergens (Halim et al., 2014, Moro et al., 2010). Type 1 and type 2 immunity are known to tightly counter-regulate each other (Stehle et al., 2016). Thus, it is not surprising that Th1 cytokines such as IFNγ antagonize the function of ILC2 and type 2 innate immune responses (Duerr et al., 2016, Moro et al., 2016). More recently, it has been reported that in the early stage of papain-induced lung inflammation in mice, depletion of NK cells resulted in increased numbers and cytokine production of ILC2s, suggesting that NK cells negatively regulate ILC2s (Bi et al., 2017). A similar mutual balance between type 1 and type 2 immunity may exist in which ILC2 counter-regulate type 1 immune responses. In line with this, Halim et al. (2016) demonstrated that IL13 production by ILC2 is crucial for licensing dendritic cells to potentiate memory Th2 cell responses in type 2 allergic diseases of the lung and skin. Thus, the lack of ILC2 and ILC2-derived cytokines may lead to a disinhibition of type I-driven immune responses such as the TNCB-induced CHS responses. How the lack of other ILC subsets such as ILC1 and ILC3 alone influences CHS reactions remains to be elucidated. A tissue protective role of ILC2-derived amphiregulin has been reported in models of respiratory infection with mouse-adapted H1N1 influenza virus and dextran sodium sulfate-induced intestinal damage (Monticelli et al., 2011, Monticelli et al., 2015). Thus, the lack of amphiregulin-mediated tissue protection may additionally promote proinflammatory effects in CHS.

Finally, an IL-10-producing ILC2 effector cell population has recently been described in murine lung and suggested to regulate immune responses in a papain-induced allergic lung inflammation model (Seehus et al., 2017). We therefore addressed a potential role of IL-10-producing ILC2s in CHS by using IL-10 transcriptional reporter mice (IL10GFP, VERTX) in the CHS model. As demonstrated previously (Dolch et al., 2017), hapten challenge of sensitized mice induced prominent IL-10 expression in Lin^+^ cells of the skin, which was almost exclusively restricted to different T-cell subsets. In contrast, no relevant IL-10 signal was detected in Lin^–^ cells in the skin of CHS or naive mice (data not shown), suggesting that at least under the conditions used in this study IL10 production of ILC2s does not play a relevant role in regulating the immune response in CHS.

Taken together, this work characterizes all ILC subsets during the inflammatory response in a TNCB-based CHS model and demonstrates that the lack of all ILC subsets or ILC2 alone leads to a markedly increased inflammatory response supporting the concept of a counter-regulatory role for ILC2s in this type 1-driven allergic skin disease.

## Material and Methods

### Mice

CD90.1 wild-type (B6.PL-Thy1/Cy) and CD90.2 *Rag1*^*–/–*^ (B6.129S7-*Rag1*^tm1Mom^) mice were maintained and/or bred in specific pathogen-free facilities at the Center for Experimental Models and Transgenic Services at the University Medical Center Freiburg. *Eomes*^Gfp/+^ x *Rorc(γt)-Cre*^Tg^ x *Rosa26R*^Yfp/+^ mice (Arnold et al., 2009, Klose et al., 2014), *Il1*5^–/–^ mice (Kennedy et al., 2000) as well as *Rora*^sg/flox^*Il7r*^Cre/+^ mice (Oliphant et al., 2014) were likewise bred in a specific pathogen-free facility. All mice were on the C57BL/6 genetic background. Experiments were performed with age- and sex-matched mice within each experiment. Age of mice ranged from 6 to 12 weeks. All of the experimental procedures were in accordance with institutional, state, and federal guidelines on animal welfare. The animal experiments were approved by the Regierungspräsidium Freiburg and supervised by the Animal Protection Representatives of the University Medical Center Freiburg or carried out with the approval of the UK Home Office, respectively (*Rora*^sg/flox^*Il7r*^Cre/+^).

### Contact hypersensitivity

For sensitization, mice (CD90.1 wild type, CD90.2 *Rag1*^*–/–*^, *Rora*^sg/flox^*Il7r*^Cre/+^, *Eomes*^Gfp/+^ x *Rorc(γt)-Cre*^Tg^ x *Rosa26R*^Yfp/+^ or *Il1*5^–/–^) were treated with epicutaneous application of 100 μl TNCB (3% w/v in acetone/olive oil) or acetone/olive oil (3 parts/1 part) alone as vehicle control to the shaved abdominal skin. Five days after sensitization, the initial ear thickness was measured, using a pocket thickness gauge (Mitutoyo, Kawasaki, Japan). After the measurement, all mice were challenged by epicutaneous application of 20 μl 1% TNCB on both ears. The ear thickness was measured every 24 hours up to 144 hours after the challenge, and the increase in ear thickness was expressed as the difference between the values before and after the challenge.

### Adoptive transfer of contact hypersensitivity and antibody-mediated ILC depletion

For the passive (adoptive transfer) CHS model, CD90.1 C57BL/6 mice were sensitized as described above. Five days after sensitization, the mice were killed, all skin draining LNs were collected, and a single-cell suspension was prepared. Cells were sorted for all T-cell subsets (CD3ε^+^CD4^+^CD8α^+^) by FACS. T cells (5 × 10^6^) were transferred by intravenous injection to naïve recipient CD90.2 *Rag1*^–/–^ mice. One hour after the injection, mice were sensitized with 20 μl of TNCB (1% w/v in acetone) on the ears. After 7 days, recipient mice were boosted (100 μl TNCB 3%w/v in acetone to the shaved abdominal skin). Finally, 21 days after T-cell transfer mice were challenged on the ears with 20 μl of TNCB (1% w/v in acetone) and the increase in ear thickness was measured as described above. For ILC depletion, mice received either 200 μg of anti-CD90.2 (clone 30-H12, Bio X Cell, West Lebanon, NH) or rat rIgG2b isotype control antibody (clone LTF-2, Bio X Cell) intraperitoneally every other day four times until 1 day before hapten challenge.

### Tissue preparation and cell isolation

Ears were collected from mice, weighed, and cut into small pieces. Subsequently, 500 μl digestion buffer, that is, Hank’s balanced salt solution containing Hepes, DNAse I, fraction II, and Liberase TM and a magnetic stirring bar, was added to every vial. Vials were put on a magnet stirrer and ears were digested for 60 minutes at 37°C. Single-cell suspensions were obtained by passing the ear digest or total LNs through a 30-μm cell strainer after which they were analyzed by flow cytometry.

### Flow cytometry

Single-cell suspensions were stained with combinations of fluorescently conjugated monoclonal antibodies (for details, see Supplementary Methods). For intracellular staining of transcription factors, cells were surface stained with a combination of antibodies, fixed, and permeabilized as recommended by the manufacturer (Thermo Fisher Scientific, Waltham, MA).

For measurement of intracellular cytokine expression, isolated cells were stimulated with phorbol 12-myristate 13-acetate (50 ng/mL, Sigma-Aldrich GmbH, Taufkirchen, Germany) and ionomycin (1,000 ng/ml, Sigma-Aldrich) or cell culture medium alone in the presence of brefeldin A (10 μg/ml, Sigma-Aldrich) and monensin (10 μg/ml Sigma-Aldrich) for 4 hours at 37°C, 5% CO_2_. Cells were surface stained with a combination of antibodies, fixed and permeabilized with a commercially available kit (BD Bioscience, Heidelberg, Germany), and stained with phycoerythrin cyanin 7-conjugated anti-IL-17A (eBio 17B17, Thermo Fisher Scientific), allophycocyanin-conjugated anti-IL5 (TRFK5, BioLegend, San Diego, CA), anti-TNFα (MP6-XT22, BD Biosciences), phycoerythrin-conjugated anti-IL22 (Poly5164, BioLegend), and anti-IFNγ (XMG1.2, Thermo Fisher Scientific). Data were acquired on a BD FACS Canto II (BD Bioscience) and analyzed using FlowJo Flow Cytometry Analysis Software (v9.5.2, Tree Star Inc., Ashland, OR).

### Statistics

Results are shown as mean ± standard deviation for single experiments or mean ± standard error of the mean when at least three independent experiments were performed. Statistical analysis was carried out using GraphPad Prism version 5.01 (GraphPad Software, La Jolla, CA) using the two-tailed, nonparametric Mann-Whitney test unless otherwise stated. Statistical significance was noted as *P < 0.05, **P < 0.01, ***P < 0.001, and ****P < 0.0001.

## Conflict of Interest

ANJM is in receipt of grant funding from the MRC (U105178805), Wellcome Trust (100963/Z/13/Z), GSK, and AstraZeneca/MedImmune. The rest of the authors state no conflict of interest.
