# Supplementary material for: Lack of Type 2 Innate Lymphoid Cells Promotes a Type I-Driven Enhanced Immune Response in Contact Hypersensitivity
Source: J Invest Dermatol. 2018 Sep;138(9):1962–72. doi: 10.1016/j.jid.2018.03.001 (PMC6117454; doi:10.1016/j.jid.2018.03.001)
Supplement: Supplementary Data [file mmc1.pdf]

SUPPLEMENTARY MATERIAL

Fig. S1

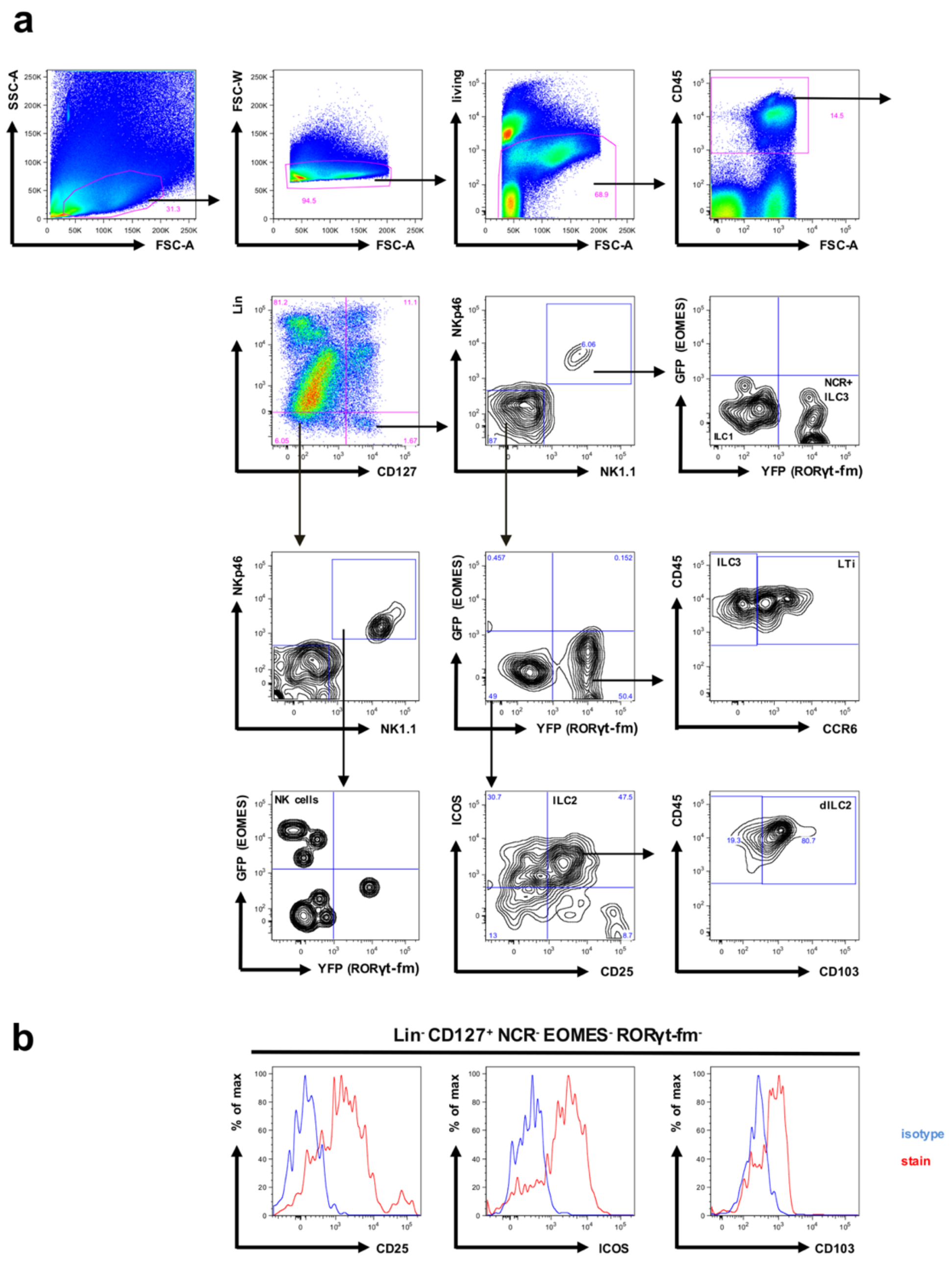

Figure S1: Gating strategy to identify ILC subclasses in EOMES^{Gfp} RORγt-fm double reporter mice and characterization of dermal ILC2. (a) For ILC gating living CD45^{+} ear skin and ear draining lymph node cells are gated for lineage negativity. NK cells are defined as CD127^{-} NCR^{+} EOMES^{+} RORγt-fm^{-}. ILC1 are gated for CD127^{+} NCR^{+} EOMES^{-} RORγt-fm^{-}. CD127^{+} NCR^{-} EOMES^{-} RORγt-fm^{-} ICOS^{+} CD25^{+} cells are defined as ILC2. ILC3 were defined as CD127^{+} NCR^{-} EOMES^{-} RORγt-fm^{+} CCR6^{-} cells. NCR^{+}ILC3 were identified as CD127^{+} NCR^{+} EOMES^{-} RORγt-fm^{+}. Finally, LTi were gated CD127^{+} NCR^{-} EOMES^{-} RORγt-fm^{+} CCR6^{+}. Lineage cocktail for identification of NK cells ILC1, ILC3, NCR^{+}ILC3 and LTi contained antibodies against the following antigens: CD5, CD19, FcεRIa, Ly-6G, F4/80, CD3ε. For ILC2 gating anti-NK1.1 antibody was added to the lineage gate. (b) ILC2 from the skin show high expression of CD25, ICOS and CD103, i.e. dermal ILC2 (dILC2). GFP reports EOMES and YFP reports RORγt-fm expression in cells. Lin, lineage; NCR, natural cytotoxicity triggering receptor, i.e. NK1.1 and NKp46; GFP, green fluorescent protein; YFP, yellow fluorescent protein. LTi, lymphoid tissue inducer cells.

Fig. S2

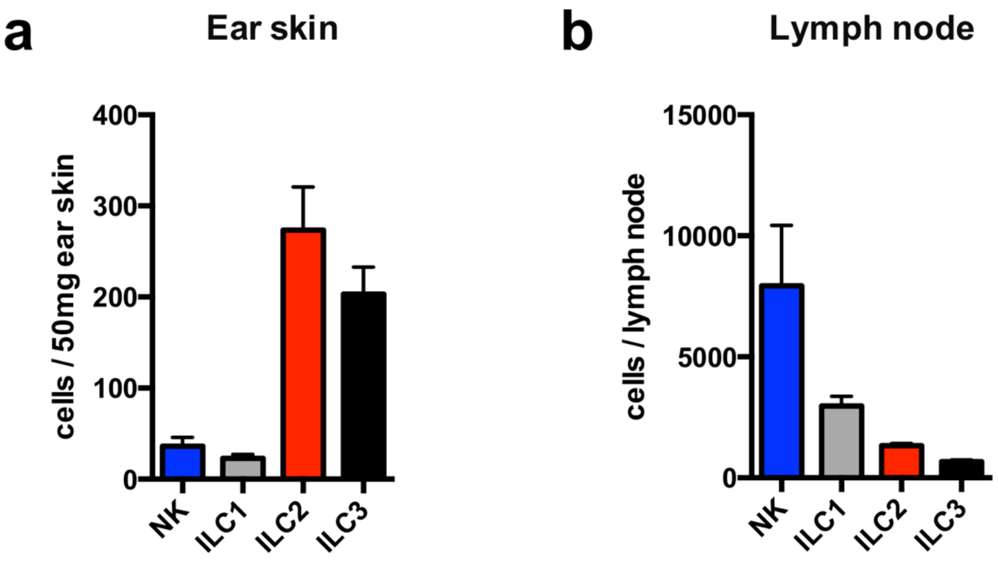

Figure S2: Total ILC numbers in mice under steady state conditions. Total ILC numbers in the ear skin (a) and ear draining lymph nodes (b) of naïve EOMES^{Gfp} RORγt-fm double reporter mice. Values are shown as absolute cell numbers per 50mg ear skin. Data are shown as mean ± standard error of the mean, pooled data of 3 independent experiments with at least n≥5 mice per group.

Fig. S3

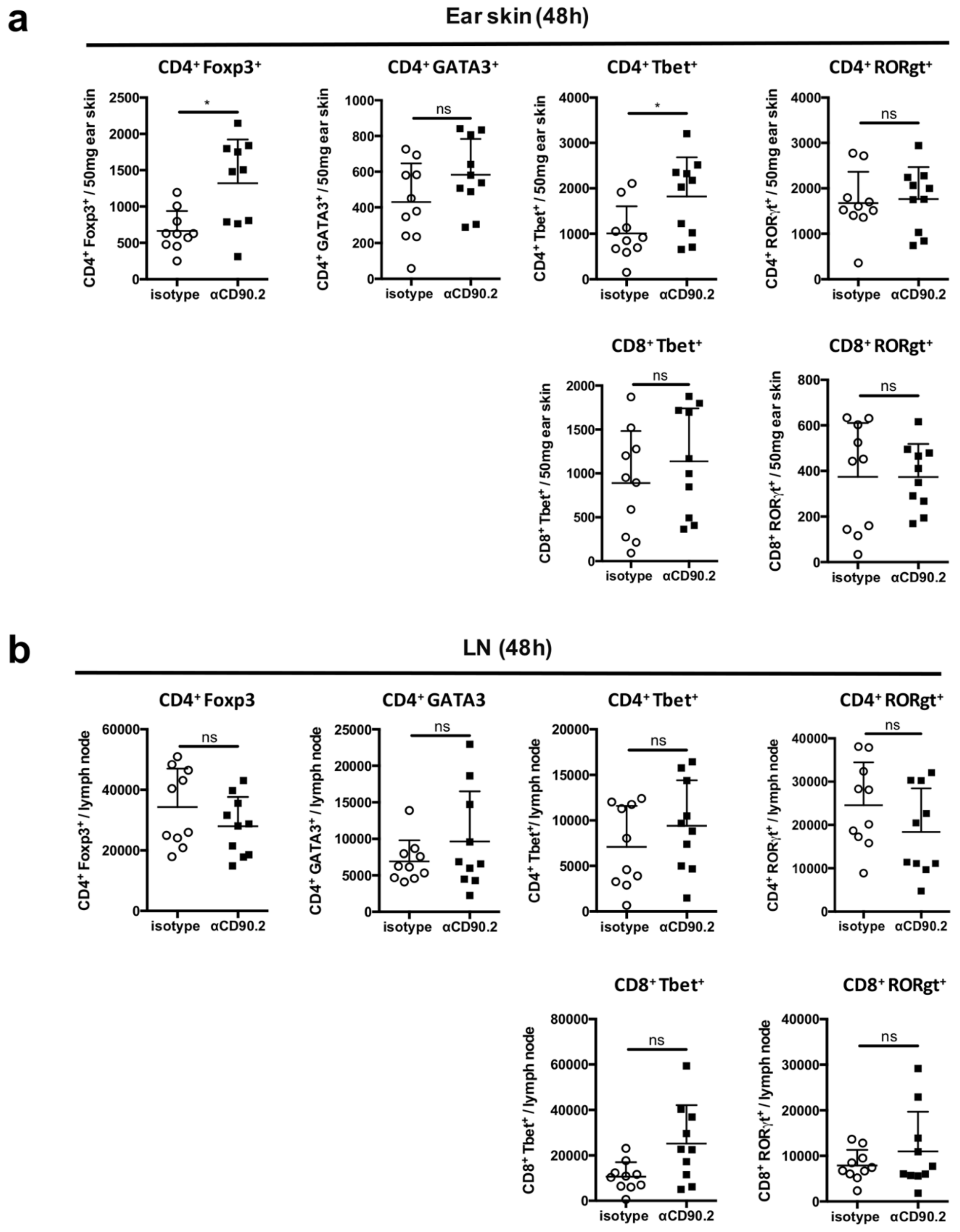

Figure S3: Total ILC depletion leads to higher numbers of T-bet and Foxp3 expressing CD4$^+$ T cells infiltrating the ear skin after allergen challenge. Total numbers of CD3$^+$ T cells expressing the transcription factors T-bet and RORγt (in CD4$^+$ and CD8$^+$ T cells) as well as Foxp3 and GATA3 (in CD4$^+$ T cells) infiltrating the ear skin (a) or the draining lymph nodes (b) of isotype and anti CD90.2 treated mice at 48h after hapten challenge. Data are shown as mean ± standard deviation. One experiment with n=10 mice per group. Values are displayed as absolute cell numbers per 50 mg ear skin and total ear draining lymph nodes, respectively. *P<0,05, **P<0,01, ***P<0,001 and ****P<0,0001, ns=not significant.

Fig. S4

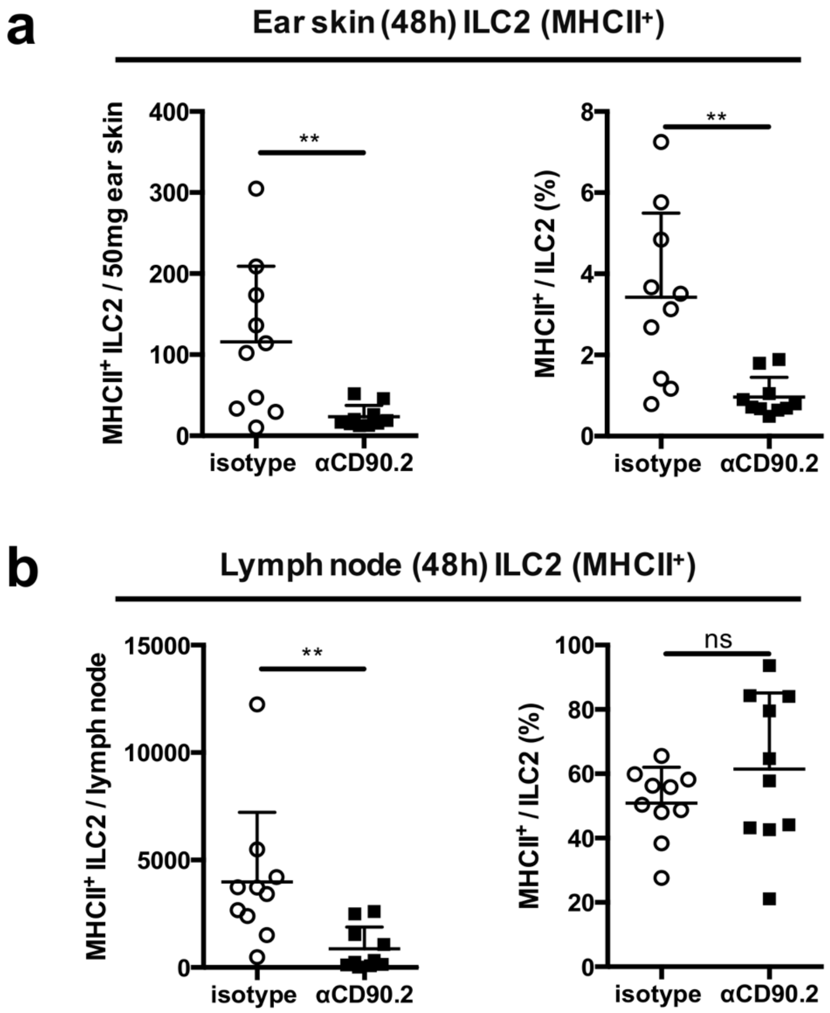

Figure S4: Antibody mediated ILC depletion leads to a significant reduction in MHCII expressing ILC2 in the skin. Total numbers of MHCII expressing ILC2 in skin (a) and lymph nodes (b) of ILC depleted vs isotype treated animals after adoptive transfer of T cells into CD90.2 *Rag1*$^{-/-}$ recipient mice and allergen challenge (for details see methods section). Values are displayed as absolute cell numbers per 50 mg ear skin and total ear draining lymph nodes, respectively. Data are shown as mean ± standard deviation, data of one experiment with n=10 mice per group. *P<0,05, **P<0,01, ***P<0,001 and ****P<0,0001, ns=not significant. CHS=contact hypersensitivity. MHCII = major histocompatibility complex class II.

Fig. S5

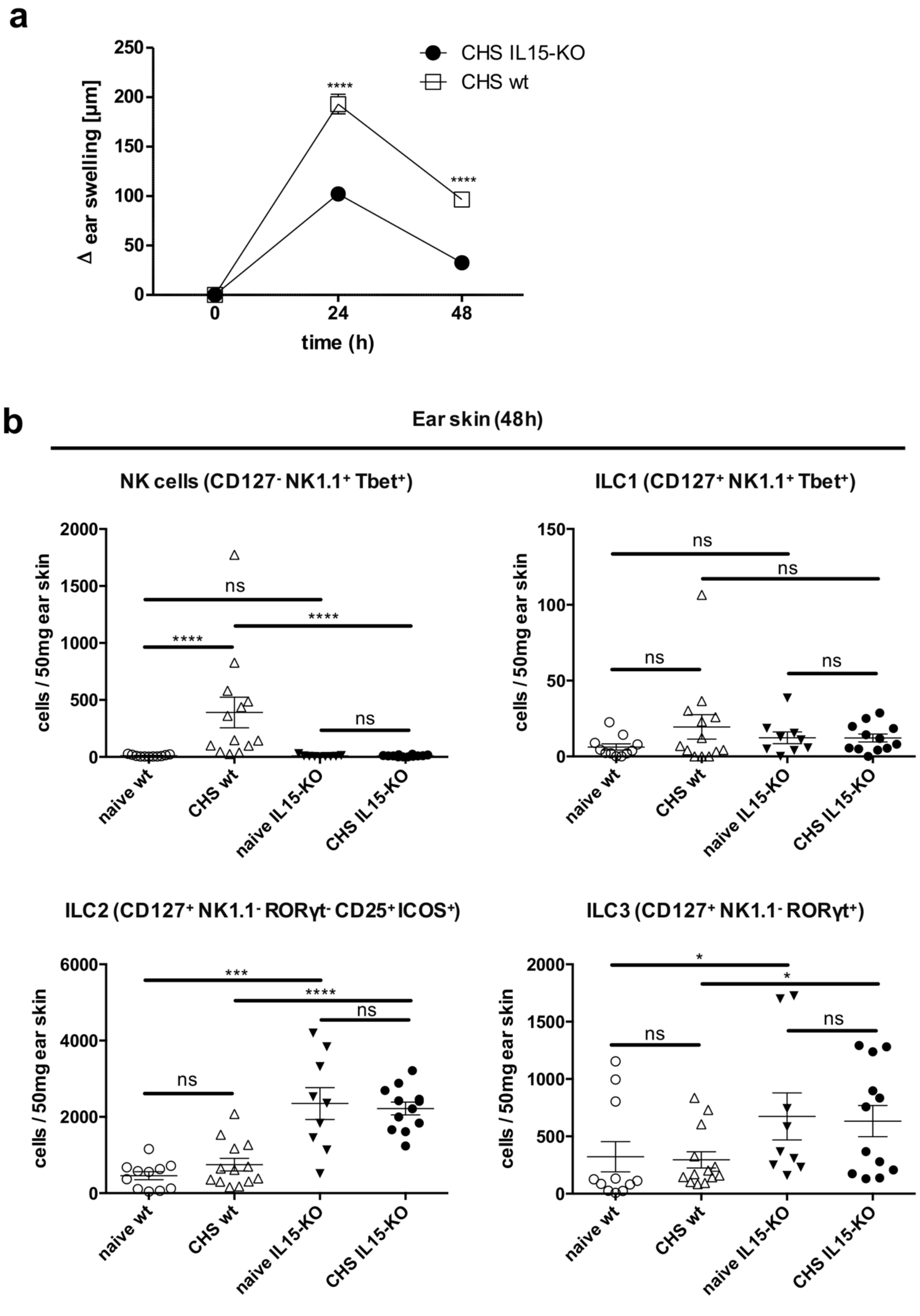
a
CHS IL15-KO
CHS wt
****
****
Δ ear swelling [µm]
time (h)
b
Ear skin (48h)
NK cells (CD127- NK1.1+ Tbet+)
ILC1 (CD127+ NK1.1+ Tbet+)
ILC2 (CD127+ NK1.1- RORγt- CD25+ ICOS+)
ILC3 (CD127+ NK1.1- RORγt+)
cells / 50mg ear skin
naive wt
CHS wt
naive IL15-KO
CHS IL15-KO
ns
*
***
****

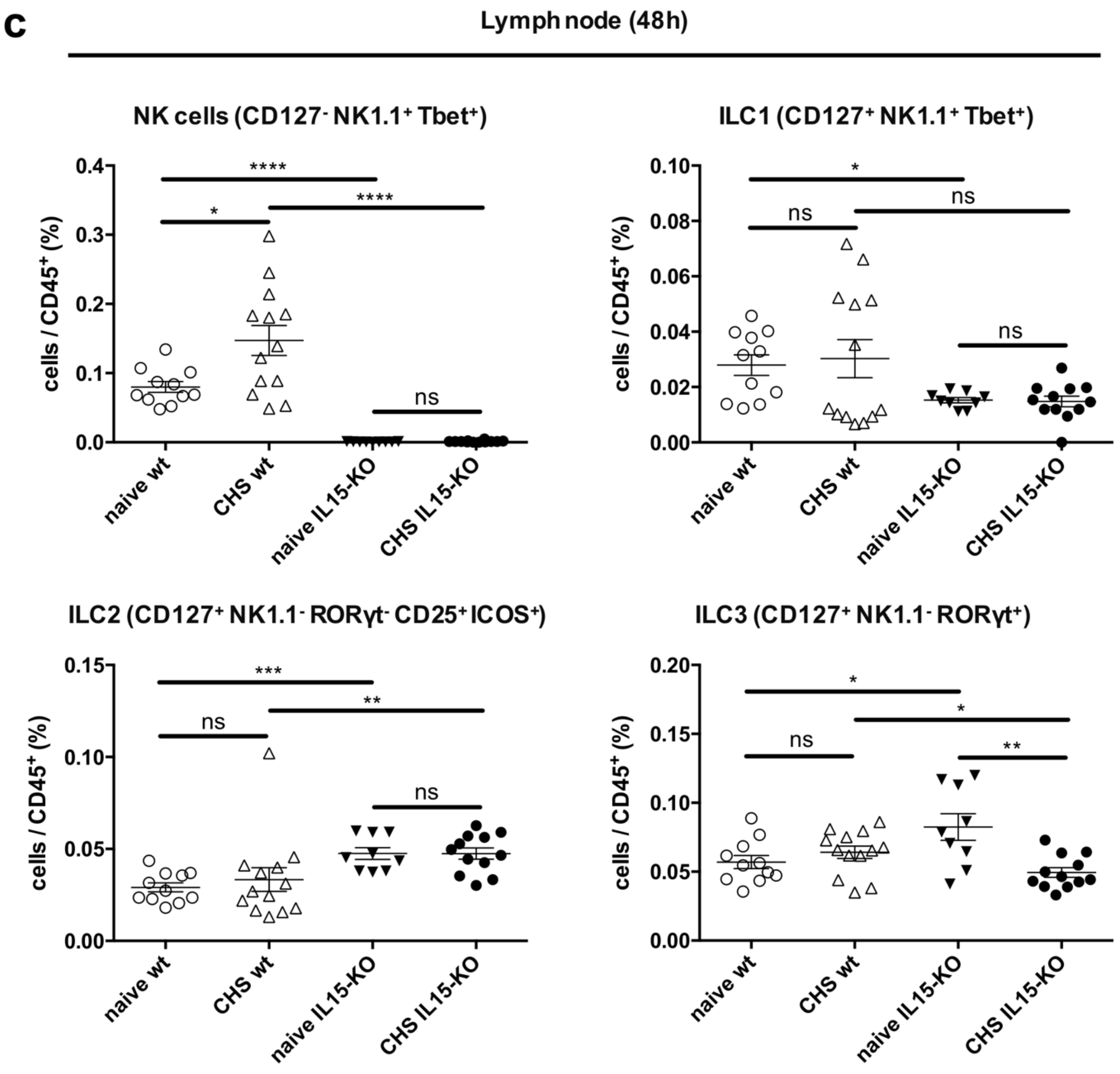

Figure S5: IL15-KO mice lacking NK cells show a decreased inflammatory response upon allergen challenge and increased numbers of ILC2 and ILC3. (a) Ear swelling response 24 and 48h after allergen challenge in IL15-KO vs. WT mice shown as delta from baseline ear thickness. Numbers of the different ILC subsets in the skin (b) and draining lymph nodes (c) of IL15-KO vs. WT mice under naïve and CHS conditions. Data are shown as mean ± standard error of the mean of 2 experiments with n≥5 mice per group. Values are displayed as absolute cell numbers per 50 mg ear skin and and percentages of living $CD45^+$ leukocytes in ear draining lymph nodes, respectively. *P<0,05, **P<0,01, ***P<0,001 and ****P<0,0001, ns=not significant.

SUPPLEMENTARY METHODS

Flow Cytometry

Single cell suspensions were stained with combinations of the following fluorescently conjugated monoclonal antibodies: biotin conjugated anti-CD3ε (145-2C11; BD Bioscience, Heidelberg, Germany), anti-CD5 (53-7.3; Biolegend, San Diego, CA), anti-CD19 (1D3, BioLegend), anti-FceRIa (MAR1, Biolegend), anti-Ly-6G (IA8, BioLegend), anti-NK1.1 (PK136, BioLegend), anti-F4/80 (BM8; Thermo Fisher Scientific, Waltham, MA), anti-CD8a (53-6.7; BD Bioscience). Phycoerythrin (PE)-conjugated anti- CD19 (1D3, Thermo Fisher Scientific), anti- anti-FcERIa (MAR1, Thermo Fisher Scientific), anti- GR-1 (RB6-8C5, BD Biosciences), anti- CD103 (2E7, Thermo Fisher Scientific), anti- IL-13 (eBio13A, Thermo Fisher Scientific), anti- CD335 (NKp46) (29A1.4, Thermo Fisher Scientific), anti- CD127 (A7R34, BioLegend), anti- CD3ε (eBio500A2, Thermo Fisher Scientific), Phycoerythrin Cyanin 7 (PE-Cy7)-conjugated anti-NK1.1 (PK136, BioLegend), anti- SCA1 (E13-161.7, BioLegend), anti-CD278 (ICOS) (C398.4A, BioLegend), anti-CD4 (GK1.5, Thermo Fisher Scientific), anti- CD117 (2B8, Thermo Fisher Scientific), anti-CD8a (53-6.7, Thermo Fisher Scientific), peridinin chlorophyll protein complex: Cy5.5 (PerCP-Cy5.5)-conjugated anti-ST2/IL-33R (RMST2-33, Thermo Fisher Scientific), anti-CD335 (29A1.4, Thermo Fisher Scientific), anti-CD3ε (145-2C11, Thermo Fisher Scientific), anti- Ly6G (HK1.4, Thermo Fisher Scientific), Allophycocyanin (APC)-conjugated anti-CD25 (PC61.5, BioLegend), anti-CD49b (DX5, BD Biosciences), anti-CCR6 (29-2L17, BioLegend), APC-eFluor780-conjugated anti CD45 (30-F11, Thermo Fisher Scientific), Pacific Blue-conjugated anti- SCA1 (D7, BioLegend), anti-CD103 (2E7, BioLegend), fluorescein isothiocyanate (FITC)-conjugated anti-CD90.2 (53-2.1 and 30-H12, BioLegend), anti-CD90.1 (HIS51, BD Biosciences), anti-T1/ST2 (DJ8, MD Bioproducts, Zürich, Switzerland), anti-CD4 (RM4-5, BD

Biosciences), PerCP-Cy5.5-conjugated Streptavidin (Thermo Fisher Scientific), eFluor 450-conjugated Streptavidin (Thermo Fisher Scientific).

Lineage cocktail for identification of NK cells ILC1 and ILC3 contained antibodies against the following antigens: CD5, CD19, FcεRIa, Ly-6G, F4/80, CD3ε. For ILC2 gating anti-NK1.1 was added to the lineage gate.

ILCs have been described to undergo changes in plasticity under certain conditions (Bernink et al. 2015; Lim et al. 2016; Melo-Gonzalez and Hepworth 2017; Zhang et al. 2017). Especially so called "ExILC3" which lose their RORγt expression and upregulate T-bet and NCRs finally leading to IFNγ production have been studied widely. However, by using $EOMES^{Gfp}$ RORγt-fm double reporter mice together with the surface markers CCR6, NK1.1 and NKp46 we were able to clearly distinguish the different ILC3 subtypes, i.e. ILC3, NCR^{+}ExILC3 and LTi cells (see also fig S1).

For intracellular staining of transcription factors, cells were surface stained with a combination of antibodies listed above, fixed and permeabilized with a commercially available kit (Thermo Fisher Scientific) and stained with PE-conjugated anti-GATA3 (TWAJ, Thermo Fisher Scientific), peridinin chlorophyll protein complex: Cy5.5 (PerCP-Cy5.5)-conjugated anti-RORγt (B2D, Thermo Fisher Scientific), Allophycocyanin (APC)-conjugated anti- FoxP3 (FJK-16s, Thermo Fisher Scientific) and anti-T-bet (eBio4B10, Thermo Fisher Scientific).

For measurement of intracellular cytokine expression, isolated cells were stimulated with phorbol 12-myristate 13-acetate (PMA) (50 ng/mL, Sigma-Aldrich GmbH, Taufkirchen, Germany) and ionomycin (1000ng/ml, Sigma-Aldrich) or cell culture medium (RPMI 1640 containing L-Glutamin (5 mM), Penicillin/Streptomycin (50 U/ml), HEPES (5 mM), β-Mercaptoethanol (0,05 mM), FBS (10 %), MEN-NAA) alone in the presence of Brefeldin A (10µg/ml, Sigma-Aldrich) and Monensin (10µg/ml Sigma-Aldrich) for 4 h at 37°C, 5% CO_2.

Cells were surface stained with a combination of antibodies listed above, fixed and permeabilized with a commercially available kit (BD Bioscience), and stained with Phycoerythrin Cyanin 7 (PE-Cy7)-conjugated anti-IL-17A (eBio 17B17, eBio4B10, Thermo Fisher Scientific), Allophycocyanin (APC)-conjugated anti-IL5 (TRFK5, BioLegend), anti-TNFα (MP6-XT22, BD Biosciences), Phycoerythrin (PE)-conjugated anti-IL22 (Poly5164, BioLegend) and anti- IFNγ (XMG1.2, eBio4B10, Thermo Fisher Scientific). Sample data were acquired on a BD FACS Canto II (BD Bioscience, Heidelberg, Germany) and analysed using FlowJo Flow Cytometry Analysis Software (v9.5.2, Tree Star Inc., Ashland, USA).

SUPPLEMENTARY REFERENCES

Bernink JH, Krabbendam L, Germar K, de Jong E, Gronke K, Kofoed-Nielsen M, et al. Interleukin-12 and -23 Control Plasticity of CD127+ Group 1 and Group 3 Innate Lymphoid Cells in the Intestinal Lamina Propria. Immunity. 2015; 43:146–60

Lim AI, Menegatti S, Bustamante J, Bourhis LL, Allez M, Rogge L, et al. IL-12 drives functional plasticity of human group 2 innate lymphoid cells. J. Exp. Med. 2016; 213:569-83

Melo-Gonzalez F, Hepworth MR. Functional and phenotypic heterogeneity of group 3 innate lymphoid cells. Immunology. 2017; 150:265–75

Zhang K, Xu X, Pasha MA, Siebel CW, Costello A, Haczku A, et al. Cutting Edge: Notch Signaling Promotes the Plasticity of Group-2 Innate Lymphoid Cells. J. Immunol. 2017; 198:1798-1803
